# Deep neural network-based clustering of deformation curves reveals novel disease features in *PLN* pathogenic variant carriers

**DOI:** 10.1007/s10554-023-02924-9

**Published:** 2023-08-11

**Authors:** Karim Taha, Rutger R. van de Leur, Melle Vessies, Thomas P. Mast, Maarten J. Cramer, Nicholas Cauwenberghs, Tom E. Verstraelen, Remco de Brouwer, Pieter A. Doevendans, Arthur Wilde, Folkert W. Asselbergs, Maarten P. van den Berg, Jan D’hooge, Tatiana Kuznetsova, Arco J. Teske, René van Es

**Affiliations:** 1 Department of Cardiology, University Medical Center Utrecht, Heidelberglaan 100, 3584 CX Utrecht, The Netherlands; 2 Netherlands Heart Institute, Utrecht, The Netherlands; 3 Informatics Institute, University of Amsterdam, Amsterdam, The Netherlands; 4 Department of Cardiology, Catharina Ziekenhuis, Eindhoven, The Netherlands; 5 Research Unit Hypertension and Cardiovascular Epidemiology, KU Leuven Department of Cardiovascular Sciences, University of Leuven, Leuven, Belgium; 6 Heart Center, Department of Cardiology, Amsterdam University Medical Center, Location Academic Medical Center, Amsterdam, The Netherlands; 7 Department of Cardiology, University Medical Center Groningen, University of Groningen, Groningen, the Netherlands; 8 Central Military Hospital, Utrecht, The Netherlands; 9 Health Data Research United Kingdom and Institute of Health Informatics, University College London, London, UK; 10 Laboratory on Cardiovascular Imaging and Dynamics, KU Leuven, Leuven, Belgium

**Keywords:** Deep learning, Clustering, Deformation imaging, Strain, Phospholamban, Cardiomyopathy

## Abstract

Echocardiographic deformation curves provide detailed information on myocardial function. Deep neural networks (DNNs) may enable automated detection of disease features in deformation curves, and improve the clinical assessment of these curves. We aimed to investigate whether an explainable DNN-based pipeline can be used to detect and visualize disease features in echocardiographic deformation curves of phospholamban (*PLN*) p.Arg14del variant carriers. A DNN was trained to discriminate *PLN* variant carriers (n = 278) from control subjects (n = 621) using raw deformation curves obtained by 2D-speckle tracking in the longitudinal axis. A visualization technique was used to identify the parts of these curves that were used by the DNN for classification. The *PLN* variant carriers were clustered according to the output of the visualization technique. The DNN showed excellent discriminatory performance (C-statistic 0.93 [95% CI 0.87–0.97]). We identified four clusters with *PLN*-associated disease features in the deformation curves. Two clusters showed previously described features: apical post-systolic shortening and reduced systolic strain. The two other clusters revealed novel features, both reflecting delayed relaxation. Additionally, a fifth cluster was identified containing variant carriers without disease features in the deformation curves, who were classified as controls by the DNN. This latter cluster had a very benign disease course regarding development of ventricular arrhythmias. Applying an explainable DNN-based pipeline to myocardial deformation curves enables automated detection and visualization of disease features. In *PLN* variant carriers, we discovered novel disease features which may improve individual risk stratification. Applying this approach to other diseases will further expand our knowledge on disease-specific deformation patterns.

**Graphical abstract:**

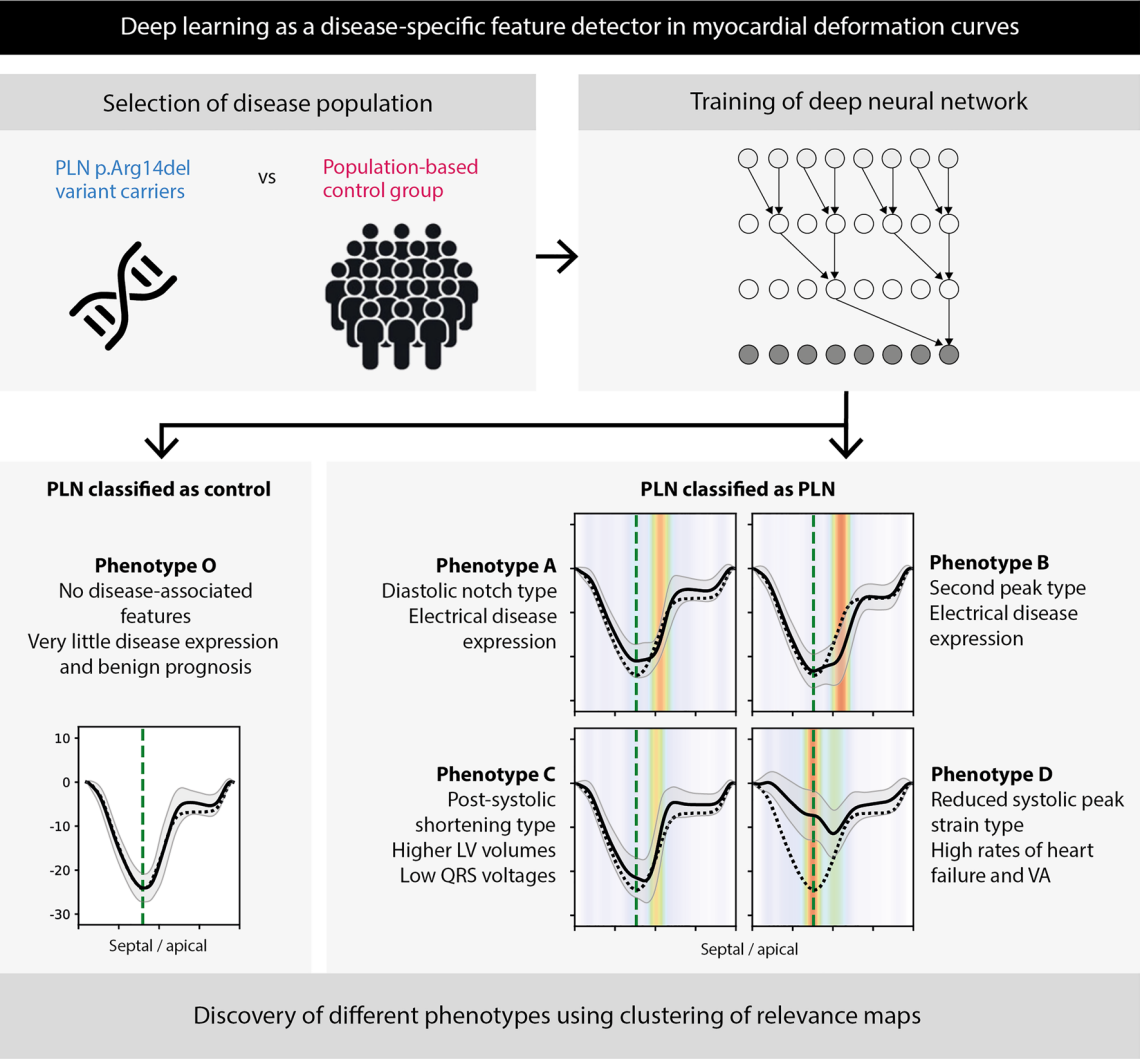

Overview of the deep neural network-based pipeline for feature detection in myocardial deformation curves. Firstly, phospholamban (*PLN*) p.Arg14del variant carriers and controls were selected and a deep neural network (DNN) was trained to detect the *PLN* variant carriers. Subsequently, a clustering-based approach was performed on the attention maps of the DNN, which revealed 4 distinct phenotypes of *PLN* variant carriers with different prognoses. Moreover, a cluster without features and a benign prognosis was detected.

**Supplementary Information:**

The online version contains supplementary material available at 10.1007/s10554-023-02924-9.

## Introduction

Mechanical deformation of the myocardium is the major determinant of cardiac function, and may be disrupted in a wide variety of cardiac diseases [1]. Changes in mechanical myocardial behavior are often caused by structural myocardial changes such as fibrosis and may ultimately lead to ventricular dysfunction and heart failure. Besides patients with structural alterations, abnormal deformation has also been reported in patients without any clear-cut structural disease, such as asymptomatic subjects with a pathogenic genetic variant [2, 3]. Since the mechanical alterations in such individuals are thought to reflect early problems in electro-mechanical coupling or even disturbed intracellular processes, accurate quantification of myocardial deformation may provide great insight into underlying disease processes.

Several non-invasive techniques exist for quantification of myocardial deformation, of which echocardiography-based 2D speckle tracking is now the most robust and most widely used technique [4]. The assessment of myocardial deformation by this technique currently relies on manual extraction of certain parameters from the deformation curve, such as peak strain. However, manual parameter extraction may lack sensitivity, especially for subtle disease processes where peak strain values fall within normal limits. Within a deformation curve, more valuable data may be concealed that we are not yet aware of [5]. An automatic method for detection of disease features in the entire deformation curve would potentially enable better characterization of (subtle) mechanical disease processes, leading to improved detection of early disease and better risk stratification.

Deep neural networks (DNNs) are computer algorithms based on the structure and function of the human brain. Their hidden layers of neurons can be trained to discover complex patterns in signals such as deformation curves. DNNs are increasingly applied to electrocardiograms (ECGs), which has led to both classification of ECGs with very high diagnostic accuracy and detection of novel ECG features [6, 7]. To date, DNNs have not been applied to deformation curves, while the deformation output features are suitable for these analyses in a similar fashion as the ECG. We hypothesize that assessment of regional deformation curves by a DNN-based approach will provide insight into spatiotemporal disease features in the deformation curve which are not yet detected by the manual approach. Discovering novel patterns by such an automated approach could greatly enhance assessment of deformation curves in routine clinical practice, potentially improving (early) disease detection and individual risk stratification of patients.

In the present study, we developed an explainable DNN-based pipeline for classification of myocardial deformation curves. As a disease model, we included subjects with the pathogenic phospholamban (*PLN*) p.Arg14del variant, who are at high risk of developing dilated and/or arrhythmogenic cardiomyopathy (DCM/ACM) [8]. All these subjects are descending from one single founder from the northern part of the Netherlands and have an identical haplotype [9]. In a previous study, we observed that regional post-systolic shortening in the left ventricular (LV) apical segments is a typical early deformation pattern in these subjects [3]. In advanced disease stages, we observed that global cardiac function becomes impaired and peak strain values are reduced. The goal of the current study was to investigate whether a DNN-based approach can be used to identify novel disease features that are concealed in the regional myocardial deformation curves of subjects with this particular genetic variant.

## Methods

### Data source and study participants

As described previously [3, 10], we selected *PLN* p.Arg14del variant carriers from a nationwide registry who underwent transthoracic echocardiography between 2006 and 2019 in the University Medical Center Utrecht, University Medical Center Groningen and Amsterdam University Medical Center. These were both index patients and family members who were identified by genetic screening. While index patients underwent comprehensive genetic testing for cardiomyopathy-related variants, family members underwent targeted testing for the *PLN* p.Arg14del variant as part of cascade family screening. Index patients with a second pathogenic cardiomyopathy-related variant and subjects with relevant cardiovascular comorbidities such as hypertension were excluded. As defined previously, *PLN* p.Arg14del variant carriers were classified as pre-symptomatic in case they had no history of ventricular arrhythmias (VA), a premature ventricular complex (PVC) count < 500/24 h and left ventricular ejection fraction (LVEF) > 45% [3].

Controls were derived from the Flemish Study on Environment, Genes and Health Outcomes (FLEMENGHO), which consists of a random population sample from a geographically defined area in Belgium [11]. The prevalence of the *PLN* p.Arg14del variant in this area was assumed to be negligible, as the prevalence of this founder variant decreases considerably towards the south [9]. We selected participants who underwent transthoracic echocardiography between 2005 and 2009 [12].

**Fig. 1:**
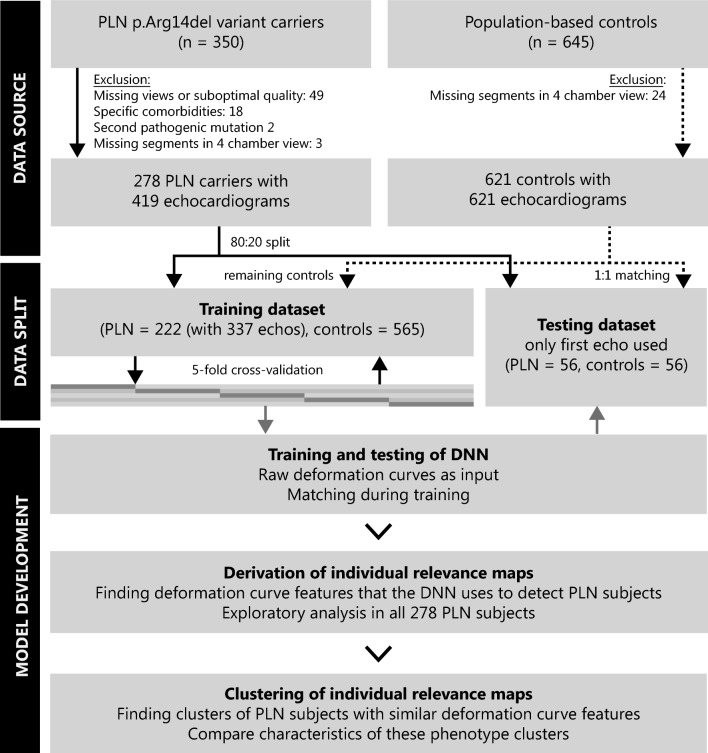
Methodology. *PLN* p.Arg14del variant carriers and control subjects were included for this study. After development of the DNN, the relevance maps of the *PLN* variant carriers were derived and the variant carriers were clustered on the basis of these relevance maps

For the training and testing of the algorithm, the *PLN* dataset was split in an 80:20 ratio on the subject level, to make sure no subjects appeared in both datasets (Fig. 1). To maximize the available data for training, multiple echocardiograms per subject were included when available. In the testing dataset, only the first echocardiogram was used, since follow-up echocardiograms may be more affected and may therefore bias the results of diagnostic performance. Every *PLN* subject in the testing dataset was matched to a control subject using propensity score matching without replacement on age, sex and heart rate, since those parameters may significantly affect the deformation curves. All remaining control subjects were eligible for the training dataset. Propensity score matching was performed during training in a 1:3 *PLN* to control ratio to account for imbalances in the previously mentioned parameters.

### Data acquisition and preprocessing

All data used in this study were retrospectively acquired data. The data were anonymized and handled according to the European General Data Protection Regulation. Re-use of the data was permitted by the Medical Ethics Committee. For the *PLN* variant carriers, the echocardiograms were acquired using a Vivid 7, E9 or E95 machine (GE Healthcare, Horten, Norway) as part of routine clinical care. For the FLEMENGO control group, echocardiograms were acquired using a Vivid 7 machine by a single experienced physician [12]. Longitudinal strain analysis was performed with 2D speckle tracking by two operators using EchoPAC software (version 2.0.3., GE Healthcare) according to the current recommendations [13]. Only apical four-chamber views were used in this analysis and were divided in six segments: apical, mid and basal segments from both the lateral and septal walls. The six regional deformation curves that were computed by the software were included in the deep learning model as raw data and normalized in time to 1 s, with the location of the aortic valve closure at 38% of the RR-interval (mean of the training data). Additional information of the data acquisition and preprocessing can be found in the Supplemental Methods.

### Model development and validation

We constructed a deep convolutional neural network with exponentially dilated causal convolutions, which is optimized for use on time series such as electrocardiograms (ECGs) [6]. By using dilated causal convolutions, the network can learn complex patterns across large segments of the waveform, while only regarding previous timepoints and therefore taking into account the temporal nature of the signal. The architecture has been described in more detail in the Supplemental Methods and an overview of the architecture can be found in Supplemental Fig. 1. Hyperparameters (i.e., number of feature maps, depth of the network, kernel size, dropout ratio, learning rate and batch size) were optimized in the training dataset using fivefold cross-validation, where complete *PLN*-control match groups were kept together during the dataset split. The simplest network with the highest F1 score averaged over all five cross-validation folds was selected for testing. The independent test dataset was used once to assess the performance of the final optimized model. To estimate this final performance, all five trained networks (from the five different cross-validation splits) were used as an ensemble model, where the probability for each subject was obtained by taking the mean of these five models’ predicted probability.

### Model visualization and feature identification

To identify the parts of the strain curves that were considered important by the model to classify *PLN* variant carriers, we used the Integrated Gradients visualization technique [14]. This approach was combined with SmoothGrad-Squared, since recent reports have shown this to be the most robust to produce individual relevance maps [15]. As phenotypical variability was expected for the *PLN* variant carriers, we performed a time series clustering approach on the relevance maps of the correctly predicted patients (i.e. with a predicted probability over 50%) in the complete dataset. K-means clustering with the Euclidean distance metric was used to divide the normalized relevance maps into four different phenotype clusters. The clusters were visualized by taking the mean and standard deviation in the temporal axis of all patients in that cluster and superimposing the cluster centers of the relevance maps as a heatmap. A Savinsky-Golay filter was applied to each cluster center to smoothen the heat map. The number of clusters was defined empirically by visually assessing whether the clusters showed difference in their morphology, while keeping a minimum of 30 patients per cluster. The mean deformation curve per segment of all the control subjects was used as a reference in the figures. Additional information on the visualization technique can be found in the Supplemental Methods.

### Follow-up data

To explore the disease course among the different phenotype clusters, follow-up data of *PLN* variant carriers were derived from an electronic research data platform [10]. The primary outcome variable was sustained VA, which was defined as sudden cardiac arrest/ventricular fibrillation, appropriate implantable cardioverter defibrillator (ICD) intervention or any recorded sustained ventricular tachycardia (> 100 bpm) lasting more than 30 s.

### Statistical analysis

Baseline data are expressed as mean ± standard deviation (SD) or median with interquartile range (IQR), where appropriate. Discriminatory performance of the deep learning model was assessed using the area under the receiver operating curve (AUC) or C-statistic, accuracy, F1-score, specificity and sensitivity. The 95% confidence intervals were derived using 2000 bootstrap samples. For the comparison of clinical characteristics between phenotype clusters, we performed Chi-square, one-way ANOVA or Kruskal–Wallis tests as appropriate. Adjustment for multiple testing was performed using Bonferroni’s correction. The DNN-based classification of the deformation curves into clusters was compared to the manual classification of the deformation curves of this cohort in a previous paper [3]. All statistical analyses were performed using Python version 3.8 (Python Software Foundation).

## Results

### Study population

Overall, 278 *PLN* variant carriers were included, with a total of 419 echocardiograms (mean of 1.5 echocardiograms per patient). From the FLEMENGO cohort, 621 control subjects were included, with one echocardiogram per subject. Baseline characteristics of the *PLN* variant carriers and control subjects (stratified by training and test set) can be found in Table 1.

**Table 1:** Baseline characteristics of the PLN mutation carriers and control subjects

	Training dataset				Testing dataset				
	Overall	Control	PLN	*P* value	Overall		Control	PLN	*P* value
n	787	565	222		112		56	56	
Echocardiograms	902	565	337		112		56	56	
Age (years)	53.1 [39.4–63.8]	55.6 [47.2–65.6]	40.1 [26.9–54.7]	< 0.001	46.9 [34.5–57.0]		48.5 [34.2–56.3]	43.7 [34.5–57.4]	1.000
Male sex	414 (52.6)	310 (54.9)	118 (53.2)	0.257	43 (38.4)		21 (37.5)	22 (39.3)	1.000
Heart rate (bpm)	61.5 ± 10.9	58.5 ± 9.6	66.4 ± 11.1	< 0.001	66.6 ± 9.7		66.4 ± 9.7	66.9 ± 9.9	1.000
Hypertension	298 (37.9)	298 (52.7)	0 (0)	< 0.001	24 (21.4)		24 (42.9)	0 (0)	< 0.001
Heart failure history	27 (3.4)	5 (0.9)	22 (9.9)	< 0.001	8 (7.1)		0 (0)	8 (14.3)	0.042
LVEF (%)	58.4 [54.1–62.0]	59.6 [56.1–63.2]	56.0 [50.0–60.0]	< 0.001	56.5 [52.4–60.8]		58.8 [55.8–62.6]	54.0 [46.5–60.0]	< 0.001
GLS (%)	18.8 ± 3.1	19.3 ± 2.3	17.9 ± 3.9	< 0.001	18.4 ± 3.8		19.7 ± 2.2	17.1 ± 4.6	0.003

Data are presented as n (%), mean ± SD or median [IQR] as appropriate. The test set is propensity score matched on age, sex and ventricular frequency in a 1:1 ratio. The training set is only propensity score matched on these variables during training. *Bpm*, beats per minute; *GLS*, global longitudinal strain; *HR*, heart rate; *LVEF*, left ventricular ejection fraction

### Performance of deep learning algorithm

Cross-validated mean C-statistic, accuracy and F1 score on the training dataset were 0.93 ± 0.02, 0.93 ± 0.01, and 0.86 ± 0.05, respectively. The performance of the ensemble model in the independent test set was excellent, with a C-statistic, accuracy, F1 score, sensitivity and specificity at a 50% probability threshold of 0.93 [95% CI 0.87–0.97], 0.90 [95% CI 0.85–0.96], 0.89 [95% CI 0.83–0.95], 0.88 [95% CI 0.79–0.96] and 0.93 [95% CI 0.85–0.98], respectively.

### Feature identification

**Fig. 2:**
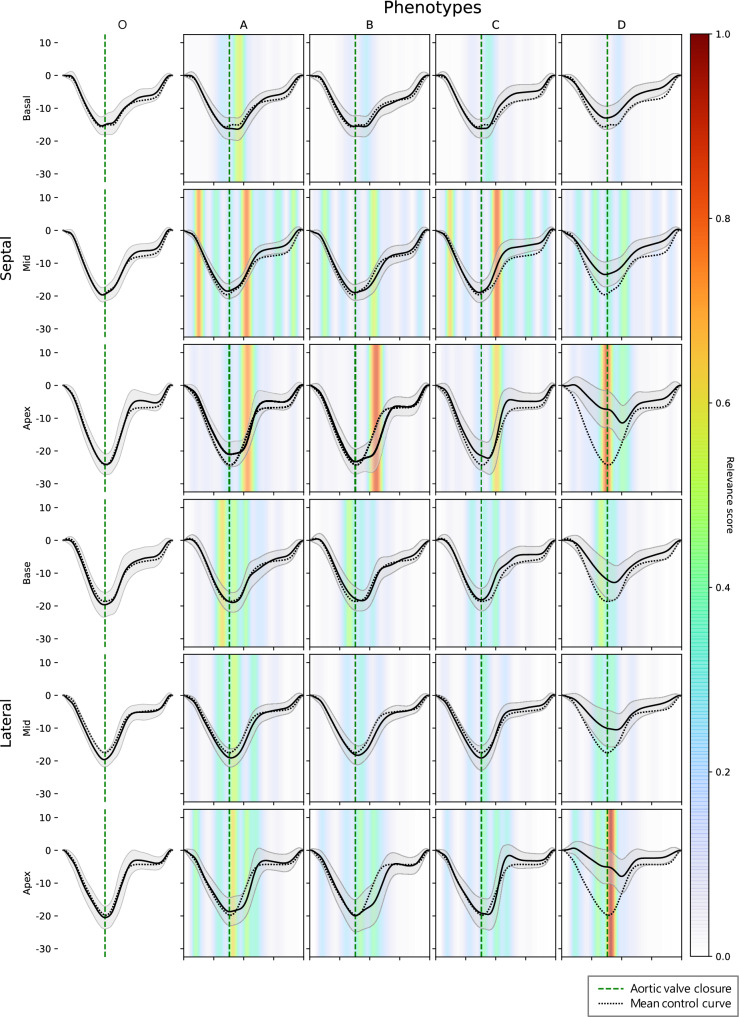
Phenotype clusters among PLN p.Arg14del variant carriers. The upper three rows represent the septal myocardial segments and the lower three rows represent the lateral myocardial segments. The solid deformation curves represent the mean deformation curve within a cluster per myocardial segment, with the standard deviation in grey. The dotted deformation curve represents the average deformation curve per segment in the control group. The vertical dotted green line represents aortic valve closure. Cluster O consists of *PLN* variant carriers who were classified by the DNN as control subjects. Clusters A–D were clustered based on the relevance maps. A higher relevance score indicates that a specific part of the deformation curve is more important for the DNN to classify someone as a *PLN* subject

Using the relevance maps generated by the Integrated Gradients visualization technique, we identified four *PLN* phenotype clusters (clusters A to D, Fig. 2). Cluster O is an additional cluster which represents the *PLN* variant carriers without any disease features in the deformation curve, who were classified as controls by the DNN (n = 27).

**Fig. 3:**
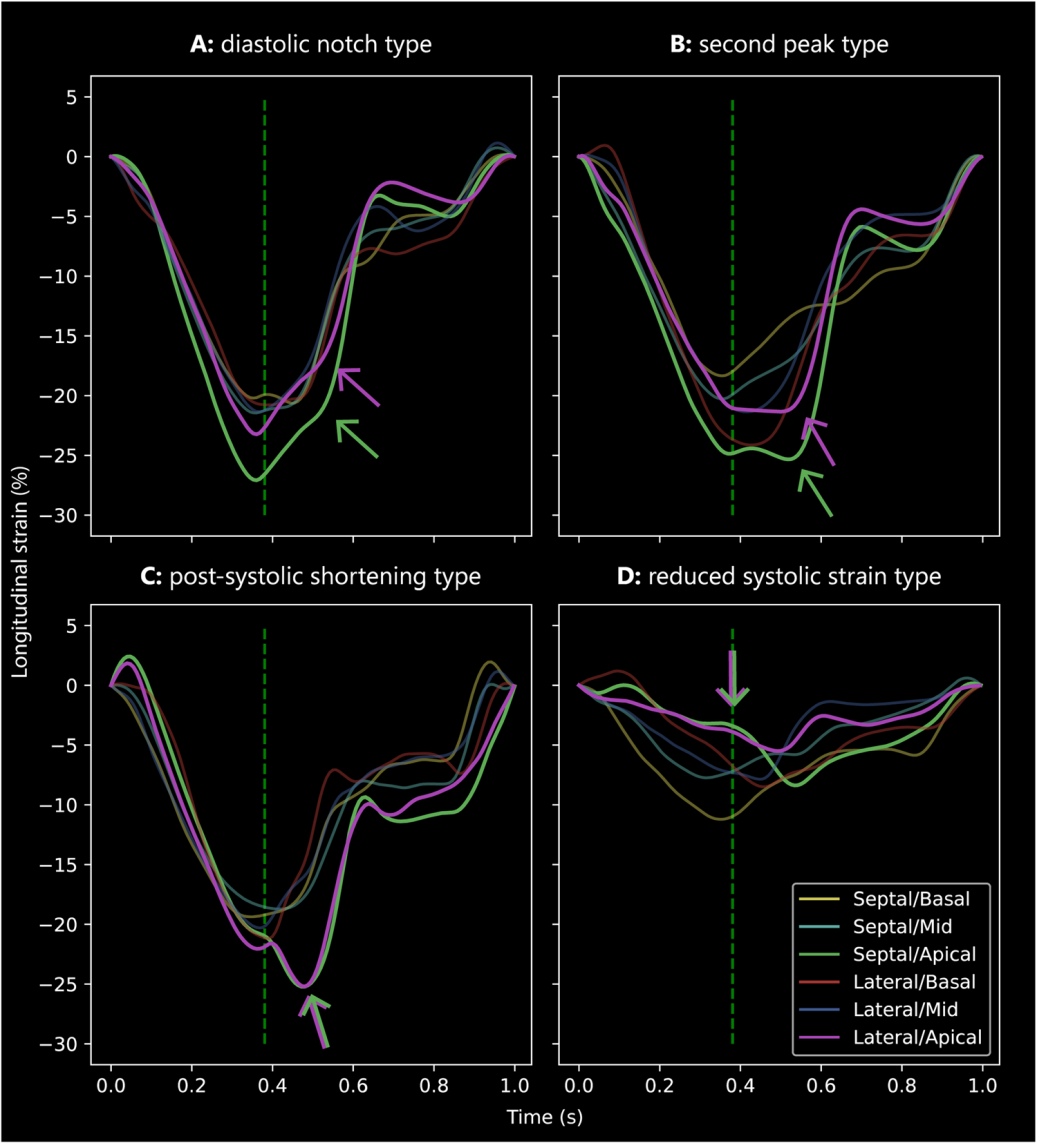
Representative examples of deformation curves from clusters A–D. The vertical green lines represent aortic valve closure. The differences between the clusters are most pronounced in the septal/lateral apical (green/purple) curves. **A** Cluster A: diastolic notch type. **B** Cluster B: second peak type. **C** Cluster C: post-systolic shortening type. **D** Cluster D: reduced systolic strain type

As shown in Fig. 2, the features that were considered most important by the DNN for classification of *PLN* variant carriers were located in the apical septal segments, mid septal segments and apical lateral segments. Temporally, these features were particularly located at end-systole and in the early diastolic phase. Figure 3 shows representative examples of subjects from each cluster. In clusters A, B and C the relevance maps demonstrated different patterns of delayed relaxation in the apical segments. In cluster A, the deformation curves consisted of one single systolic peak, with a notch during the normal upstroke of the curve after aortic valve closure in the septal apical segment (type A pattern: ‘diastolic notch’, Fig. 3A). Importantly, the deformation curves in this cluster did not show additional shortening after aortic valve closure. By manual classification, 47 of the subjects in cluster A (56%) were previously classified as having normal deformation curves in the apical segments. In cluster B, there were two peaks of shortening in the septal apical segment, of which the first peak occurred before or at aortic valve closure, and the second peak after aortic valve closure (type B pattern: ‘double peak’, Fig. 3B). By manual classification, 25 subjects in cluster B (56%) were previously classified as having normal deformation curves in the apical segments. In cluster C, the deformation curves showed pronounced post-systolic shortening in the apical segments (type C pattern: ‘post-systolic shortening’, Fig. 3C). The relevance maps in this cluster focused specifically on diastolic upstroke of the deformation curve in the apical and mid septal segment, where high diastolic strain rate values were found (Supplemental Table 1). By manual classification, 36 subjects in cluster C (54%) were previously classified as having normal deformation curves in the apical segments. The deformation curves in cluster D were characterized by decreased systolic peak strain values, which was considered most important in the septal and lateral apical segments (type D pattern: ‘reduced peak strain’, Fig. 3D). By manual classification, all subjects in cluster D were previously classified as having abnormal deformation curves in the apical segments.

### Clinical cluster characteristics

Additional clinical data of the phenotype clusters can be found in Table 2. Subjects in cluster O, who were recognized as controls by the DNN, were relatively young (36 [IQR 27–46] years), were all identified by family screening and the majority was pre-symptomatic (n = 22, 82%). Conventional echocardiographic measurements in this cluster were without exception within normal limits. Subjects in cluster A were older than subjects in cluster O (40 [IQR 24–53], but still had normal conventional echocardiographic measurements. Subjects in cluster B were older than subjects in cluster A (46 [IQR 34–64] years) with slightly reduced relaxation parameters (E-wave velocity and e’). All other conventional parameters in this cluster were preserved. The subjects in cluster A and B both had more electrical disease expression compared to cluster O with regard to T-wave inversions, low QRS voltages, VA history and PVC burden per 24 h (Table 2). Subjects in cluster C were the youngest of all clusters (36 [IQR 24–44] years), and were characterized by relatively high LV end-diastolic and end-systolic volumes (58.3 [IQR 52.9–65.8] ml/m^2^ and 24.7 [IQR 20.8–27.9] ml/m^2^, respectively) and a particularly high rate of low QRS voltages (38.1%). Finally, cluster D consisted of the oldest subjects (54 [IQR 43–61] years) with the most advanced disease, with high rates of heart failure and VA, and severely impaired conventional echocardiographic LV and RV measurements.

**Table 2:** Characteristics of the phenotype clusters

	Overall	O	A	B	C	D	*P* value
n	278	27	84	45	67	55	
Patient characteristics							
Age (years)	41.4 [28.6–54.8]	36.5 [27.4–46.7]	40.0 [23.8–52.5]	45.7 [33.5–63.6]	36.1 [24.0–44.4]	53.6 [42.9–61.1]	< 0.001
Male sex	126 (45.3)	15 (55.6)	31 (36.9)	19 (42.2)	33 (49.3)	28 (50.9)	1.0
Proband	49 (17.6)	0 (0)	12 (14.3)	2 (4.4)	9 (13.4)	26 (47.3)	< 0.001
Presymptomatic	139 (50.0)	22 (81.5)	47 (56.0)	28 (62.2)	41 (61.2)	1 (1.8)	< 0.001
History of heart failure	30 (10.8)	0 (0)	3 (3.6)	2 (4.4)	3 (4.5)	22 (40.0)	< 0.001
History of VA	95 (34.2)	4 (14.8)	22 (26.2)	9 (20.0)	15 (22.4)	45 (81.8)	< 0.001
History of sustained VA	42 (15.2)	0 (0)	7 (8.3)	1 (2.2)	4 (6.0)	30 (54.5)	< 0.001
ICD implanted	58 (21.0)	1 (3.7)	11 (13.3)	2 (4.4)	8 (11.9)	36 (66.7)	< 0.001
ACM diagnosis	50 (18.5)	0 (0)	11 (13.8)	8 (17.8)	6 (9.1)	25 (48.1)	< 0.001
DCM diagnosis	42 (15.3)	0 (0)	5 (6.1)	2 (4.4)	4 (6.1)	31 (57.4)	< 0.001
Echocardiogram characteristics							
Heart rate (bpm)	66.2 ± 11.1	69.9 ± 12.2	68.6 ± 10.2	72.1 ± 10.1	59.9 ± 9.6	63.7 ± 10.0	< 0.001
LVED volume (ml/m^2^)	56.5 [48.8–64.4]	53.7 [49.2–56.9]	53.8 [46.9–61.7]	49.4 [44.3–55.1]	58.3 [52.9–65.8]	75.3 [60.0–91.4]	< 0.001
LVES volume (ml/m^2^)	24.3 [19.5–31.6]	23.3 [19.0–24.9]	22.5 [18.3–28.4]	20.9 [18.6–23.3]	24.7 [20.8–27.9]	45.9 [34.6–63.5]	< 0.001
LVEF (%)	56.0 [50.0–60.0]	59.0 [56.0–61.0]	57.0 [52.0–60.2]	58.0 [53.0–61.0]	57.0 [55.0–60.5]	39.0 [29.0–46.0]	< 0.001
GLS (%)	19.0 [16.5–20.5]	19.4 [18.6–20.9]	19.5 [17.9–20.4]	19.6 [18.5–20.6]	19.9 [18.4–21.4]	12.1 [8.1–14.9]	< 0.001
RVED area (cm^2^/m^2^)	10.0 [8.8–11.8]	10.0 [9.5–10.9]	9.7 [8.2–10.8]	9.4 [8.2–11.1]	9.6 [9.0–11.7]	12.9 [10.2–15.6]	< 0.001
RVES area (cm^2^/m^2^)	5.7 [4.6–7.2]	5.5 [5.0–5.7]	5.4 [4.2–6.8]	5.2 [4.2–6.1]	5.2 [4.5–6.8]	9.2 [6.4–10.8]	< 0.001
FAC (%)	41.6 ± 9.1	46.9 ± 4.1	43.3 ± 7.7	45.1 ± 7.8	44.2 ± 7.2	32.0 ± 8.7	< 0.001
LVMD (ms)	40.0 [30.0–55.0]	31.0 [27.5–38.0]	37.0 [29.0–43.0]	38.0 [29.5–55.5]	35.0 [27.0–48.0]	62.5 [54.2–70.8]	< 0.001
E-wave velocity (cm/s)	72.2 (19.3)	71.4 (18.7)	76.2 (21.1)	64.0 (14.3)	78.4 (16.7)	63.1 (18.9)	0.001
A-wave velocity (cm/s)	52.0 (16.0)	55.2 (14.3)	53.4 (15.4)	58.9 (15.7)	46.1 (12.3)	49.7 (20.3)	0.024
Average e’ (cm/s)	11.4 (4.0)	11.8 (4.1)	12.2 (3.9)	10.6 (3.0)	13.4 (3.6)	6.9 (2.0)	< 0.001
E/e’	6.9 (2.7)	6.3 (1.3)	6.6 (2.3)	6.4 (1.7)	6.4 (2.9)	9.2 (3.5)	< 0.001
LAVI (ml/m^2^)	30.9 (9.5)	27.6 (7.1)	29.2 (6.9)	29.0 (7.1)	29.1 (8.5)	39.1 (12.4)	< 0.001
Other clinical investigations							
Inferolateral T-wave inversion	67 (24.1)	2 (7.4)	18 (21.4)	11 (24.4)	14 (20.9)	22 (40.0)	0.391
Low QRS voltage	85 (31.6)	3 (11.1)	15 (18.3)	8 (18.2)	24 (38.1)	35 (66.0)	< 0.001
PVC amount (per 24 h)	178.0 [4.0–1574.0]	11.5 [2.5–151.5]	95.0 [3.8–1041.2]	145.0 [3.0–1172.0]	41.5 [2.0–639.2]	2185.0 [1099.8–5065.0]	< 0.001

Data are presented as n (%), mean ± SD or median [IQR] as appropriate. *P* values are derived by one-way Analysis of Variance. *ACM*, arrhythmogenic cardiomyopathy; *bpm*, beats per minute, *DCM*, dilated cardiomyopathy; *FAC*, fractional area change; *ICD*, implantable cardioverter defibrillator; *LAVI*, left atrial volume index; *LVED/LVES*, left ventricular end diastolic/systolic; *LVEF*, left ventricular ejection fraction; *LVMD*, left ventricular mechanical dispersion; *PVC*, premature ventricular complex; *RVED/RVES*, right ventricular end diastolic/systolic; *VA*, ventricular arrhythmia

### Follow-up data

**Fig. 4:**
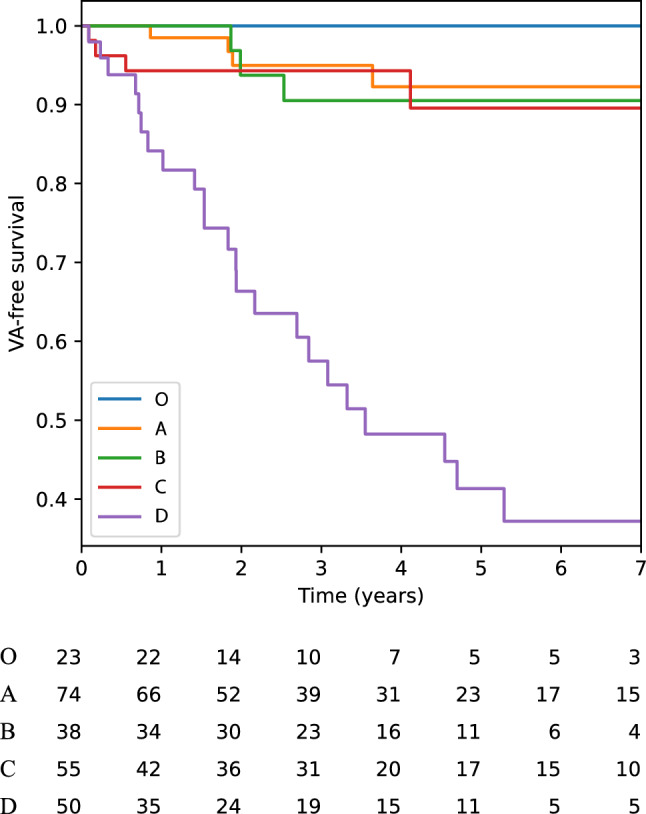
VA-free survival among the five phenotype clusters. Follow-up data was available for 240/278 *PLN* variant carriers (86%). Overall, sustained VA occurred in 34 patients during follow-up. Subjects in cluster O had the most benign prognosis (none of them experienced VA), whereas subjects in cluster D had the worst prognosis. The prognosis was similar among subjects in clusters A, B and C

Follow-up data was available for 240/278 *PLN* variant carriers (86%). During a median follow-up duration of 3.0 years [IQR 1.4–5.2 years], 34 patients (14%) experienced the sustained VA endpoint. These were 4 (5%) from cluster A, 3 (8%) from cluster B, 4 (7%) from cluster C and 23 (46%) from cluster D. None of the subjects that were allocated to cluster O experienced a sustained arrhythmia during follow-up. Figure 4 shows the Kaplan–Meier curves for this endpoint, stratified by the phenotype clusters.

## Discussion

This study is the first one to use a DNN for discovery of novel disease features in myocardial deformation curves. By using a completely novel pipeline for deformation curves that combines an explainable DNN with a clustering approach, we were able to (i) identify five distinct phenotype clusters among *PLN* p.Arg14del variant carriers, (ii) confirm previously described disease features which are characteristic for this genetic variant, and (iii) discover novel disease features that were, up to now, concealed in the deformation curves of these variant carriers. These novel features can be used to reclassify deformation curves that were previously considered normal. Importantly, the phenotype clusters identified by this approach seem to have distinct prognostic differences, which could potentially lead to improvement of individual risk stratification. Applying this approach to other patient populations will likely enrich our knowledge on deformation patterns in a broad variety of other diseases.

### Automated detection of disease features

Myocardial deformation curves contain a large amount of information on intrinsic mechanical myocardial properties. However, the interpretation of deformation curves is challenging, which hampers the routine clinical use of these curves. Previously, attempts have been made to classify deformation curves by extracting certain parameters manually and applying disease-specific cut-off values [2]. However, this approach is complicated by the fact that strain values may fall within normal limits during early stages of disease, and peak strain values are influenced by variety of parameters such as pre- and afterload. Instead, assessment of the entire deformation curve to detect disease-specific patterns is probably more appropriate [5]. However, the knowledge of such disease-specific patterns is currently limited. Therefore, we investigated the utility of DNN-based classification combined with an advanced visualization technique to detect and visualize disease features in deformation curves. While current DNN visualization techniques usually only provide insight on the individual subject level, we used a clustering approach to describe different phenotype clusters among the investigated disease population.

### Disease features in PLN variant carriers

This study was specifically designed to explore the unique strain characteristics in patients with a homogeneous genetic background. In a recent observational study, we found that post-systolic shortening in the LV apex is a typical deformation pattern in *PLN* p.Arg14del variant carriers who are in early stages of disease [3]. In more advanced stages of disease, we observed a global reduction of peak strain. These deformation patterns were now also detected by our DNN-based approach, and are shown in cluster C and D, respectively. The DNN-based approach expanded our knowledge by showing that the reduction of peak strain in cluster D is most pronounced in the apical segments. Post-systolic shortening was also present in this cluster, but the DNN did not consider it to be of additional value on top of the reduced peak strain in the apical segments.

Strikingly, the results of this study also added novel data on specific diastolic myocardial behavior that has not been recognized before. In clusters A and B, we observed specific patterns of delayed relaxation, where overt post-systolic shortening is not (yet) present. It is conceivable that these disease features in early diastole can be explained by the pathophysiological mechanism of the *PLN* p.Arg14del variant, since it is thought that this variant causes *PLN* to inhibit calcium reuptake into the sarcoplasmic reticulum, leading to a diastolic calcium overload in the cardiomyocyte [16].

Since disease mechanisms vary among carriers of different variants, we assume that the identified features are specific for this *PLN* variant. In future studies, it would be of great interest to include patients with a broad variety of variants into a DNN, to characterize variant-specific disease features and personalize diagnostic protocols for carriers of all different variants. This was beyond the scope of the present explorative study.

Since the prognostic value for developing VA was similar among clusters A, B and C, one could argue that there is no clinical relevance in distinguishing these clusters. However, it is important to note that by manual classification, a significant part of the deformation curves from clusters A, B and C would be classified as normal (56%, 56% and 54%, respectively). With our approach, we could reclassify the deformation curves from these individuals from normal to abnormal. This highlights the clinical relevance of our approach, considering the difference in development of VA between the normal cluster O and the abnormal clusters A, B and C.

### PLN variant carriers without disease features

Clusters A to D all contained *PLN* variant carriers who were correctly classified by the DNN. In addition, we described cluster O which contained variant carriers who were not recognized by the DNN as variant carriers due to the absence of disease features. It is known that the *PLN* p.Arg14del variant has age-related penetrance with symptoms often beginning around the fifth decade, which implies that there is a pre-phenotypical phase in which disease expression is absent [8]. Therefore, it is conceivable that the subjects in cluster O are the ones who still lack disease expression, and who can therefore not be distinguished from population controls by the DNN. This is supported by the fact that subjects in this cluster were mostly young, pre-symptomatic family members who were identified by family screening. The follow-up data demonstrated that the disease course in this cluster was benign; none of the subjects in cluster O developed a sustained ventricular arrhythmia during 3 years of follow-up.

### Clinical implications and future directions

Since the *PLN* p.Arg14del variant is characterized by large phenotypical variability, this novel DNN-based approach may be useful for classifying subjects with this particular variant into one of the phenotype clusters. Subjects with this variant who do not exhibit disease features (cluster O) can perhaps undergo low-frequency follow-up, whereas subjects who are classified in the most advanced disease cluster (cluster D) may possibly benefit from more intensive follow-up and appropriate therapeutic intervention, for example ICD implantation. Future studies should elaborate on the prognostic value of these clusters, also considering other clinical variables [17].

Besides using this approach for classification purposes, this approach is very useful to gain insight into characteristic disease features that are concealed in the deformation curves. Ideally, this approach should be applied to other diseases, which will expand our knowledge on disease-specific deformation patterns and potentially improve the interpretation of deformation curves in clinical practice. In this proof-of-concept study we only used the deformation curves from the apical 4-chamber view, but in future studies it would be of interest to include the deformation curves from all apical views, including the atrial and right ventricular deformation curves. Combining the deformation curves with ECG data in a DNN model would also be of interest in future studies.

### Limitations

This study has several limitations to address. First, our control group was derived from a population-based cohort, while the group of variant carriers were scanned and analyzed in other centers. This may have induced center-specific differences between the controls and the variant carriers. Second, the developed DNN was not validated in an external cohort. However, the aim of this study was not to investigate the performance of the algorithm, but to propose a novel way to detect diseases-specific features in deformation curves. Third, the visualization technique that was used in this study only shows the temporal location of important features in the deformation curve, but it does not specify what the feature exactly is. The actual description of the features was performed by visual assessment and should be validated in future studies. Fourth, since detection of regional abnormalities by deformation imaging is limited by inter-vendor variability, it remains unknown whether the results can be generalized to other vendors [18]. Fifth, the strain measurements in our study were performed by different operators. However, inter- and intra-observer agreement of several strain measurements were reported to be good in previous studies by our group [19]. Last, we reported follow-up data to explore prognostic differences among the clusters, but the number of events in this cohort was too low to perform appropriate statistical survival analyses. This remains to be investigated in future studies with longer follow-up intervals.

## Conclusion

Applying an explainable DNN-based pipeline to myocardial deformation curves allows for automated discovery of (novel) disease features. In subjects with the genetic *PLN* p.Arg14del variant, this approach detected two previously described features (i.e. apical post-systolic shortening and decreased systolic peak strain), and more importantly, revealed two novel features reflecting delayed relaxation. The different phenotype clusters seem to have distinct prognostic differences, which could lead to improvement of individual risk stratification in this group of variant carriers. Applying this novel pipeline to other patient populations will enrich our knowledge on deformation characteristics in a broad variety of diseases, which could improve the assessment of deformation curves in clinical practice.

### Supplementary Information

Below is the link to the electronic supplementary material.

Supplementary file1 (DOCX 186 KB)

## Data Availability

The datasets used in this study are not openly available due to privacy concerns. The code for training the DNN and for generating the visualizations and tables in this paper is available upon request from the corresponding author.
