# Supplementary material for: Deep neural network-based clustering of deformation curves reveals novel disease features in PLN pathogenic variant carriers
Source: Int J Cardiovasc Imaging. 2023 Aug 11;39(11):2149–61. doi: 10.1007/s10554-023-02924-9 (PMC10673970; doi:10.1007/s10554-023-02924-9)
Supplement: Supplementary file 1 — Supplementary file1 (DOCX 186 KB) [file 10554_2023_2924_MOESM1_ESM.docx]

**SUPPLEMENTAL MATERIAL**

**Supplemental Methods**

*Data acquisition and processing*

Echocardiograms were excluded if the image quality was insufficient for deformation analysis (i.e. frame rate <50/s or requiring exclusion of any segment). Since 32% of the apical three-chamber views and 37% of the apical two-chamber views had missing segments, only the apical four-chamber view was used for this study. The apical four-chamber view was automatically divided in six segments by the software: apical, mid and basal segments from both the lateral and septal walls. Timing of aortic valve closure was obtained from the B-mode images in the apical three-chamber view. The six regional deformation curves that were computed by the software were included in the deep learning model as raw data. All curves were resampled to 128Hz and normalized in the time domain to 1 second (equivalent of heart rate of 60 per minute) per patient. The time-normalization was performed using an alignment function to normalize differences in the location of the aortic valve closure due to heart rate differences (i.e. different ratio between systole and diastole). Using the time marker of the aortic valve closure, the alignment function ensured the aortic valve closure marker of each sample to be aligned with the mean aortic valve closure marker of the training data, at 38% of the RR-interval.

*Model visualization and feature identification*

To identify the parts of the strain curves that were considered important by the model to classify PLN mutation carriers, we used the Integrated Gradients visualization technique in combination with SmoothGrad-Squared.^1–5^ For each PLN subject, a temporal and per-segment relevance map was produced by approximating the integral of gradients of the output with respect to a given baseline deformation curve. To improve the robustness of this approach, this procedure was performed 200 times per subject, where a different baseline waveform was sampled from the control subjects every time and Gaussian noise was added to the patient’s waveform ^5^. The standard deviation used for the noise in the temporal axis was calculated using the curves of all control subjects, which is important since a deformation curve has less variation at the edges. The mean of the 200 squared relevance maps was subsequently used for further analysis. As we also used five different models as an ensemble, the maps were calculated for each model and averaged afterwards. Finally, extreme outliers in a single curve were clipped (i.e. values above the 99^th^ percentile in that specific curve) and the maps were normalized per patient.

*DNN model architecture and training*

We constructed a convolutional deep neural network architecture using exponentially dilated causal 1D convolutions to encode the 1D strain waveforms. This network architecture has been shown to work well with 1D median beat ECG data before and is inspired by Van den Oord et al and Bos et al.^6–8^ The network with the final hyperparameter set was first composed of four 1-dimensional causal convolutional blocks to transform the 6x128-sized strain waveform data to 16 128-dimensional feature maps.^9^ Subsequently, we employed a 1-dimensional adaptive max pooling layer to squeeze the temporal dimension resulting in a 16-dimensional representation and finally two linear layers transforming the squeezed temporal information to one output logit. Each causal convolution block consisted of a combination of causal convolutions, weight normalizations, leaky ReLUs and residual connections.^10–12^ The dilation parameter used in the convolutional layer was exponentially doubled in each subsequent causal convolution block from 1 to 16. For all 4 blocks, the number of output channels of the convolutional layers was kept constant at 16. All convolutional layers used a kernel size of 5 and a value of 0.01 was used for the negative slope parameter of the leaky ReLU activation functions. In between the two final linear layers, batch normalization, a ReLU activation function and dropout (with probability of 0.3) was performed.^13^ For training, we optimized the network parameters using a binary focal loss function to handle class imbalance, and Adam with a learning rate of 0.001 as the optimization algorithm.^14,15^ This loss function reshapes the standard binary cross entropy such that it down-weights the loss assigned to well-classified examples. The used batch size was 32. An overview of the architecture can be found in Supplemental Figure 1. All network training was performed using the Pytorch package (version 1.7).

References

1. Sundararajan M, Taly A, Yan Q. Axiomatic Attribution for Deep Networks. *Arxiv* 2017.

2. Hooker S, Erhan D, Kindermans P-J, Kim B. A Benchmark for Interpretability Methods in Deep Neural Networks. *Arxiv* 2018.

3. Adebayo J, Gilmer J, Muelly M, Goodfellow I, Hardt M, Kim B. Sanity Checks for Saliency Maps. *Advances in Neural Information Processing Systems* 2018;2018-Decem:9505--9515.

4. Ancona M, Ceolini E, Öztireli C, Gross M. Towards better understanding of gradient-based attribution methods for Deep Neural Networks. *Arxiv* 2017.

5. Smilkov D, Thorat N, Kim B, Viégas F, Wattenberg M. SmoothGrad: removing noise by adding noise. *Arxiv* 2017.

6. Oord A van den, Dieleman S, Zen H, Simonyan K, Vinyals O, Graves A, Kalchbrenner N, Senior A, Kavukcuoglu K. WaveNet: A Generative Model for Raw Audio. *Neural Comput* 2016;21:793--830.

7. Bos MN, Leur RR van de, Vranken JF, Gupta DK, Harst P van der, Doevendans PA, Es R van. Automated Comprehensive Interpretation of 12-lead Electrocardiograms Using Pre-trained Exponentially Dilated Causal Convolutional Neural Networks. *2020 Comput Cardiol* 2020;00:1–4.

8. Leur RR van de, Taha K, Bos MN, Heijden JF van der, Gupta D, Cramer MJ, Hassink RJ, Harst P van der, Doevendans PA, Asselbergs FW, Es R van. Discovering and Visualizing Disease-Specific Electrocardiogram Features Using Deep Learning: Proof-of-Concept in Phospholamban Gene Mutation Carriers. *Circulation Arrhythmia Electrophysiol* 2021;14.

9. Franceschi J-Y, Dieuleveut A, Jaggi M. Unsupervised Scalable Representation Learning for Multivariate Time Series. Wallach H, Larochelle H, Beygelzimer A, Alche-Buc F d\textquotesingle, Fox E, Garnett R, eds. *Advances in Neural Information Processing Systems*. Curran Associates, Inc.; 2019. p4650--4661.

10. Salimans T, Kingma DP. Weight normalization: A simple reparameterization to accelerate training of deep neural networks. *Advances in Neural Information Processing Systems* 2016:901--909.

11. He K, Zhang X, Ren S, Sun J. Deep residual learning for image recognition. *2016 Ieee Conf Comput Vis Pattern Recognit Cvpr* 2016;2016-Decem:770--778.

12. Maas AL, Hannun AY, Ng AY. Rectifier nonlinearities improve neural network acoustic models. *in ICML Workshop on Deep Learning for Audio, Speech and Language Processing* 2013;28.

13. Srivastava N, Hinton G, Krizhevsky A, Sutskever I, Salakhutdinov R. Dropout: A Simple Way to Prevent Neural Networks from Overfitting. *Journal of Machine Learning Research* 2014;15:1929--1958.

14. Kingma DP, Ba J. Adam: A Method for Stochastic Optimization. Bengio Y, LeCun Y, eds. *3rd International Conference on Learning Representations*. San Diego, CA, USA: Conference Track Proceedings; 2015.

15. Lin TY, Goyal P, Girshick R, He K, Dollar P. Focal Loss for Dense Object Detection. *2017 Ieee Int Conf Comput Vis Iccv* 2017;2017-Octob:2999--3007.


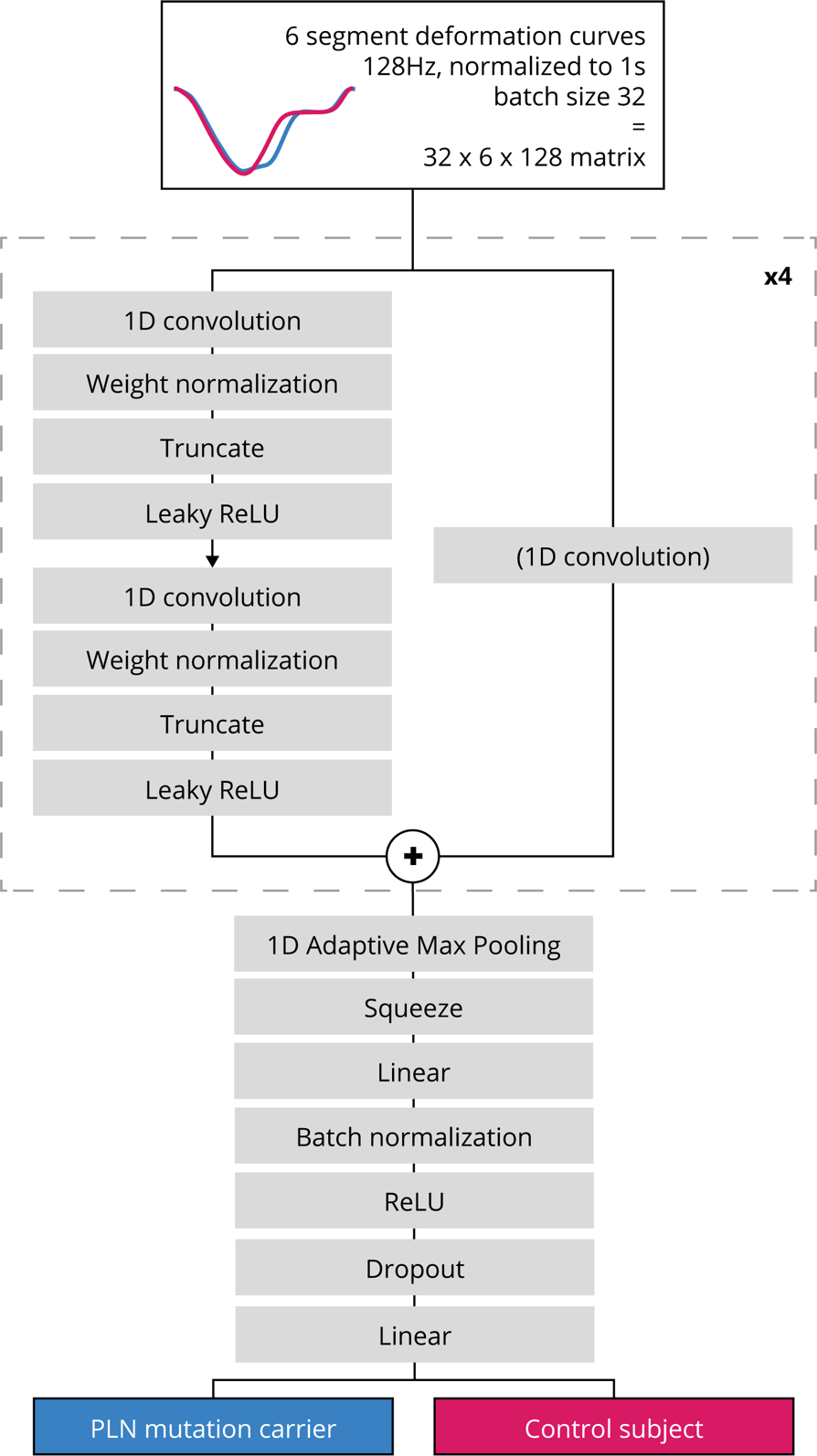


*Supplemental Figure 1.* Overview of the architecture used to construct the deep neural network for interpretation of deformation curves. A batch of size 32 x 6 x 128 is entered into the model during training. ReLU: rectified linear unit, PLN: phospholamban.

*Supplemental Table 1.* Values of several regional strain parameters in the different clusters

|  | **Overall** | **O** | **A** | **B** | **C** | **D** | **P-Value** |
| --- | --- | --- | --- | --- | --- | --- | --- |
| **n** | 278 | 27 | 84 | 45 | 67 | 55 |  |
| **Maximum strain (%)** |  |  |  |  |  |  |  |
| Septal/basal, median [IQR] | -16.6 [-18.7--14.2] | -15.3 [-17.7--14.3] | -17.4 [-19.7--14.9] | -16.8 [-18.6--14.8] | -17.2 [-18.7--15.7] | -13.7 [-16.0--10.9] | <0.001 |
| Septal/mid, median [IQR] | -18.7 [-20.4--16.4] | -20.2 [-21.0--18.5] | -19.2 [-20.2--17.6] | -19.5 [-21.5--17.3] | -19.5 [-20.6--18.1] | -14.6 [-16.5--11.5] | <0.001 |
| Septal/apical, median [IQR] | -22.2 [-25.4--19.2] | -25.2 [-26.1--22.3] | -22.2 [-24.8--20.1] | -24.8 [-27.6--22.2] | -24.1 [-26.5--21.2] | -11.6 [-17.0--9.6] | <0.001 |
| Lateral/basal, median [IQR] | -18.6 [-20.9--16.0] | -19.0 [-23.7--16.7] | -19.8 [-21.2--17.5] | -19.1 [-20.8--17.3] | -18.8 [-21.3--16.7] | -13.7 [-17.9--10.2] | <0.001 |
| Lateral/mid, median [IQR] | -18.6 [-20.6--15.7] | -19.4 [-20.4--18.0] | -19.6 [-20.9--18.1] | -18.6 [-20.6--16.5] | -19.7 [-21.6--17.4] | -11.4 [-14.9--7.1] | <0.001 |
| Lateral/apical, median [IQR] | -19.4 [-22.6--15.5] | -20.4 [-22.5--18.1] | -19.8 [-22.4--16.8] | -21.1 [-24.1--17.5] | -20.6 [-24.2--18.4] | -8.5 [-13.0--5.2] | <0.001 |
| **Maximum strain rate (%/s)** |  |  |  |  |  |  |  |
| Septal/basal, median [IQR] | 0.8 [0.6-0.9] | 0.8 [0.7-0.9] | 0.8 [0.6-0.9] | 0.7 [0.6-1.0] | 0.9 [0.7-1.1] | 0.6 [0.5-0.7] | <0.001 |
| Septal/mid, median [IQR] | 0.8 [0.7-1.0] | 0.9 [0.7-1.0] | 0.8 [0.7-0.9] | 0.8 [0.6-1.0] | 1.1 [0.8-1.2] | 0.6 [0.5-0.8] | <0.001 |
| Septal/apical, median [IQR] | 1.5 [1.1-2.1] | 1.3 [1.1-1.7] | 1.6 [1.2-1.9] | 1.5 [1.2-1.7] | 2.2 [1.6-2.7] | 0.9 [0.6-1.3] | <0.001 |
| Lateral/basal, median [IQR] | 1.0 [0.8-1.3] | 1.1 [0.9-1.2] | 1.0 [0.8-1.3] | 1.0 [0.9-1.3] | 1.2 [1.0-1.6] | 0.7 [0.5-1.0] | <0.001 |
| Lateral/mid, median [IQR] | 0.9 [0.7-1.2] | 1.0 [0.8-1.2] | 0.9 [0.8-1.2] | 0.8 [0.6-1.0] | 1.2 [0.9-1.4] | 0.6 [0.4-0.8] | <0.001 |
| Lateral/apical, median [IQR] | 1.4 [1.0-1.9] | 1.4 [0.9-1.6] | 1.5 [1.1-1.8] | 1.3 [1.1-1.5] | 2.3 [1.7-2.8] | 0.7 [0.5-1.0] | <0.001 |
| **Minimum strain rate (%/s)** |  |  |  |  |  |  |  |
| Septal/basal, median [IQR] | -0.7 [-0.7--0.6] | -0.7 [-0.8--0.6] | -0.7 [-0.8--0.6] | -0.7 [-0.7--0.6] | -0.7 [-0.8--0.7] | -0.5 [-0.6--0.4] | <0.001 |
| Septal/mid, median [IQR] | -0.7 [-0.7--0.6] | -0.7 [-0.8--0.7] | -0.7 [-0.7--0.6] | -0.7 [-0.8--0.6] | -0.7 [-0.8--0.7] | -0.5 [-0.6--0.4] | <0.001 |
| Septal/apical, median [IQR] | -0.8 [-1.0--0.7] | -0.9 [-0.9--0.8] | -0.8 [-0.9--0.7] | -0.9 [-1.0--0.8] | -0.9 [-1.0--0.8] | -0.6 [-0.8--0.5] | <0.001 |
| Lateral/basal, median [IQR] | -0.8 [-0.9--0.6] | -0.8 [-0.9--0.8] | -0.8 [-0.9--0.7] | -0.8 [-0.9--0.7] | -0.8 [-0.9--0.7] | -0.6 [-0.6--0.5] | <0.001 |
| Lateral/mid, median [IQR] | -0.6 [-0.8--0.5] | -0.7 [-0.8--0.6] | -0.7 [-0.8--0.6] | -0.7 [-0.8--0.5] | -0.7 [-0.8--0.6] | -0.4 [-0.5--0.4] | <0.001 |
| Lateral/apical, median [IQR] | -0.8 [-0.9--0.6] | -0.8 [-0.9--0.7] | -0.8 [-0.9--0.7] | -0.8 [-0.9--0.7] | -0.9 [-1.0--0.7] | -0.5 [-0.7--0.4] | <0.001 |
| **Post systolic shortening (%)** |  |  |  |  |  |  |  |
| Septal/basal, median [IQR] | -1.0 [-7.1--0.0] | -2.0 [-6.2--0.0] | -2.2 [-12.3--0.0] | -2.9 [-6.7--0.0] | -0.9 [-6.4--0.0] | -0.1 [-5.0--0.0] | 1.000 |
| Septal/mid, median [IQR] | 0.0 [-0.8--0.0] | 0.0 [-0.0--0.0] | 0.0 [-0.6--0.0] | -0.0 [-2.5--0.0] | 0.0 [-0.0--0.0] | 0.0 [-3.7--0.0] | 0.109 |
| Septal/apical, median [IQR] | -4.9 [-18.6--0.0] | -0.2 [-3.1--0.0] | -3.4 [-9.4--0.0] | -3.0 [-13.9--0.0] | -5.2 [-14.1--0.1] | -52.0 [-79.6--12.5] | <0.001 |
| Lateral/basal, median [IQR] | -3.0 [-10.0--0.0] | -2.5 [-4.3--0.0] | -2.6 [-8.4--0.1] | -6.9 [-14.8--0.3] | -0.6 [-2.6--0.0] | -9.1 [-27.5--3.8] | <0.001 |
| Lateral/mid, median [IQR] | -0.8 [-4.0--0.0] | 0.0 [-0.8--0.0] | -1.1 [-5.8--0.0] | -1.1 [-2.7--0.3] | 0.0 [-0.7--0.0] | -9.0 [-24.0--1.8] | <0.001 |
| Lateral/apical, median [IQR] | -1.8 [-19.6--0.0] | 0.0 [-2.8--0.0] | -0.9 [-11.8--0.0] | -1.2 [-7.5--0.0] | -2.0 [-12.3--0.0] | -48.5 [-95.7--6.6] | <0.001 |
